# Acetoacetate Ameliorates Hepatic Fibrosis by Targeting Peroxisome Proliferator-Activated Receptor Gamma to Restore Lipid Droplets in Activated Hepatic Stellate Cells

**DOI:** 10.3390/ph18020219

**Published:** 2025-02-06

**Authors:** Ya Zhou, Feixia Wang, Mengru Hu, Siwei Xia, Yang Li, Shizhong Zheng, Feng Zhang

**Affiliations:** Jiangsu Key Laboratory for Pharmacology and Safety Evaluation of Chinese Materia Medica, School of Pharmacy, Nanjing University of Chinese Medicine, Nanjing 210023, China; 20220821@njucm.edu.cn (Y.Z.); 300585@njucm.edu.cn (F.W.); 20230653@njucm.edu.cn (M.H.); 20223116@njucm.edu.cn (S.X.); 20200773@njucm.edu.cn (Y.L.)

**Keywords:** hepatic fibrosis, acetoacetate, PPARγ, lipid droplets

## Abstract

**Background:** Hepatic fibrosis (HF) is a progressive liver disease characterized by the activation of hepatic stellate cells (HSCs) and changes in lipid metabolism. Abnormal ketone body (KD) levels, including acetoacetate (AcAc) and beta-hydroxybutyrate (BHB), have been observed in patients with HF, but the mechanisms linking ketone metabolism to fibrosis progression remain unclear. **Objectives:** This study aimed to investigate the role of AcAc in modulating HSCs activation and its potential mechanisms in HF. **Methods:** We examined the effects of AcAc on HSCs activation by Western blot analysis and RT-PCR both in vivo and in vitro. The impact of AcAc on lipid droplet accumulation in HSCs was assessed using total cholesterol (TC), triglyceride (TG), and Retinol (RET) kits, along with Nile Red and Oil Red O staining. RT-PCR screening was performed to analyze the expression of genes involved in lipid droplet formation and lipid metabolism. **Results:** Our findings show that AcAc inhibited HSCs activation by restoring LD levels. Peroxisome Proliferator-Activated Receptor Gamma (PPARγ) was identified as a key regulator through gene screening. AcAc primarily regulated PPARγ expression, and knocking down PPARγ significantly aggravated HF progression. **Conclusions:** The ability of AcAc to restore LD levels and regulate PPARγ suggests that it may represent a promising therapeutic strategy for HF by inhibiting HSCs activation.

## 1. Introduction

Hepatic fibrosis (HF) is a pathological change caused by chronic liver injury leading to excessive deposition of extracellular matrix (ECM), and its mechanism of occurrence is related to endothelial cell injury, inflammatory immune cell activation, and hepatic stellate cells (HSCs) activation [1]. It plays a crucial role in the progression of chronic liver disease to cirrhosis and eventually to hepatocellular carcinoma [2]. Common etiologic factors include viral hepatitis, alcoholic liver disease, nonalcoholic fatty liver disease, drug toxicity, and autoimmune diseases [3,4]. The pathophysiologic process of HF involves multiple cell types and molecular mechanisms, with HSCs playing a key role in the development of fibrosis [4]. Under normal conditions, HSCs exhibit a quiescent phenotype [5]. When stimulated by hepatic injury and inflammatory responses, HSCs transform from a quiescent to a highly proliferative and contractile myofibroblast phenotype, secreting large amounts of ECM proteins, releasing a series of pro-inflammatory factors (e.g., IL-6, etc.) and pro-fibrotic factors (e.g., TGF-β1) [6], overexpressing α-SMA, fibronectin, collagen and other proteins, which promote the progression of HF [7]. Therefore, activated HSCs have been regarded as a major target for the treatment of HF.

Lipid droplets (LDs), as the main form of intracellular lipid storage, play an important role in the metabolic regulation of HSCs [8]. It was found that the release of LDs containing retinyl esters and triglyceride is a defining feature of HSC activation, and that quiescent HSCs cytoplasm is enriched in vitamin A lipid droplets, which, when activated, have a reduced capacity to store vitamin A, leading to a decrease in lipid droplet content in HSCs [9], and our previous study also demonstrated that inhibition of lipid droplet autophagy ameliorates HF by increasing lipid content in HSCs [10]. Changes in intracellular lipid droplet content are mainly influenced by the conjugation or metabolism of lipid droplets [11]. PPARγ, a key factor in the synthesis of LDs, has been shown to be closely associated with HF, but its molecular mechanism is still unclear [12,13].

As an important metabolic organ, the liver is involved in various material and energy metabolic processes [14]. Therefore, studying the pathogenesis of HF from the perspective of metabolic regulation is expected to provide new drug targets for HF and promote the development of safe and efficient antifibrotic drugs. Ketone body metabolism is a metabolic process carried out exclusively in the mitochondria of hepatocytes, which mainly includes acetoacetate (AcAc), β-hydroxybutyrate (BHB), and trace amounts of acetone [15]. AcAc is a naturally occurring metabolite produced by the liver that provides cellular energy through conversion to acetyl-CoA. It also involves conversion to other metabolites, including acetone and acetoacetate derivatives, which are ultimately excreted in the urine or exhaled [16]. Dysregulation of ketone body metabolism is closely associated with the development of several diseases [17,18]. A study has shown that targeting 3-oxoacid CoA-transferase 1 (OXCT1) to regulate ketone body metabolism in macrophages helps to reduce the depletion of CD8+ T cells, which in turn maintains their anti-tumor function and enhances immune responses [17]. In addition, the injection of AcAc was able to prevent the deposition of ECM in HF mice, suggesting that this pathway may play a role in HF; however, the exact mechanism of action is not clear [18]. Therefore, the aim of this study was to explore the mechanisms of ketone bodies AcAc and BHB in regulating HSCs activation and lipid droplet synthesis to further understand the role of ketone bodies in HF.

## 2. Results

### 2.1. The Ketone Body Acetoacetate (AcAc) but Not Beta-Hydroxybutyrate (BHB) Inhibits Hepatic Stellate Cells (HSCs) Activation

**Figure 1:**
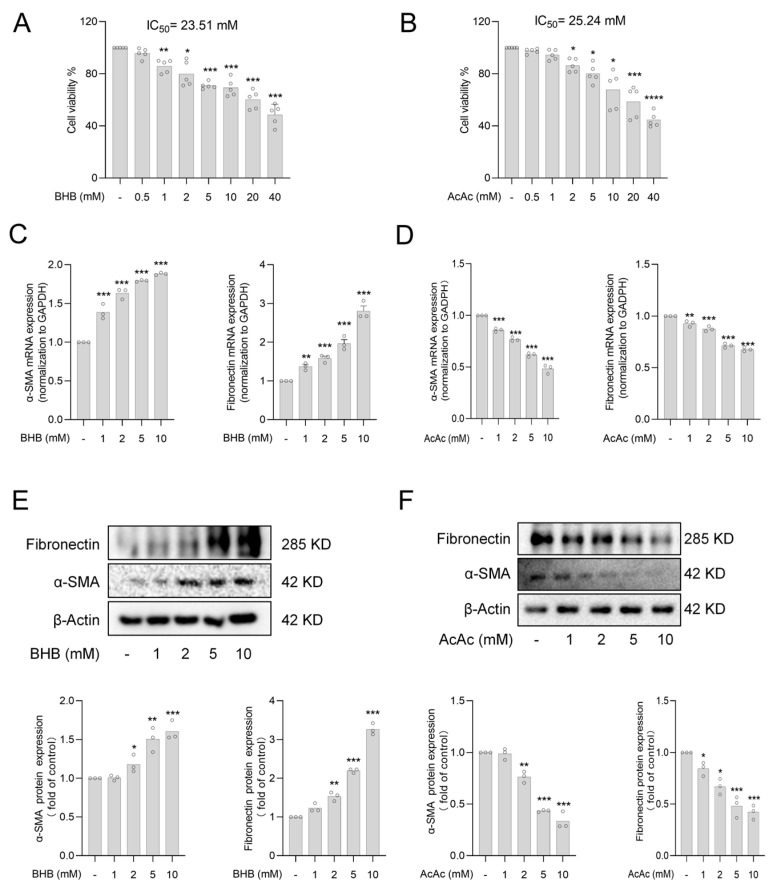
The ketone body AcAc but not BHB inhibits HSC activation. (**A**,**B**) The MTT assay was used to determine the cell viability of LX-2 after AcAc and BHB, respectively (n = 5 per group). (**C**) The mRNA levels of *α-SMA* and *fibronectin* in LX-2 cells treated with BHB were determined by RT-qPCR (n = 3 per group). (**D**) The mRNA levels of *α-SMA* and *fibronectin* in LX-2 cells treated with AcAc were determined by RT-qPCR (n = 3 per group). (**E**) Protein levels of α-SMA and fibronectin in BHB-treated LX-2 cells were determined by Western blot analysis (n = 3 per group). (**F**) Protein levels of α-SMA and fibronectin in LX-2 cells treated with AcAc were determined by Western blot analysis (n = 3 per group). Bars represent the mean ± standard error of the mean (SEM); * *p* < 0.05, ** *p* < 0.01, *** *p* < 0.001, **** *p* < 0.0001 vs. control.

Ketone bodies are composed of acetoacetic acid (AcAc), beta-hydroxybutyric acid (BHB), and acetone [15]. Since acetone has a low concentration in the blood and is mainly exhaled rather than directly utilized by cells, its metabolism in vivo is limited and therefore less of a major focus of research. Therefore, the present study focused on the role of AcAc and BHB. In order to investigate the direct effects of AcAc and BHB on hepatic stellate cells, we cultured the HSC cell line LX-2 cells, and LX-2 was treated with AcAc and BHB selected according to the available literature (0–40 mM) for 24 h [19]. The results of MTT assay showed that 2 mM of AcAc and 1 mM of BHB significantly reduced cell viability, and in order to select the concentration of drug treatments more accurately, we further calculated the IC_50_ values of the two, which were AcAc = 25.24 mM and BHB = 23.51 mM, respectively. Then, in order to rule out the toxicity effect of the drugs on the cells, we chose 1 mM, 2 mM, 5 mM, and 10 mM for the experiments (Figure 1A,B). α-SMA and fibronectin are markers of HSCs activation, and both reflect the progression of HF, which are important biomarkers for assessing HF. Therefore, we further analyzed the mRNA and protein expression of α-SMA and fibronectin by RT-qPCR and Western blot analysis after AcAc and BHB treatments, which found that AcAc was able to reduce the expression of α-SMA and fibronectin concentration-dependently, whereas BHB had the opposite effect. The results presented above demonstrated that AcAc significantly inhibited the activation of hematopoietic stem cells, whereas BHB did not exhibit such an effect (Figure 1C–F).

### 2.2. Acetoacetate (AcAc) Alleviated Hepatic Fibrosis (HF)

**Figure 2:**
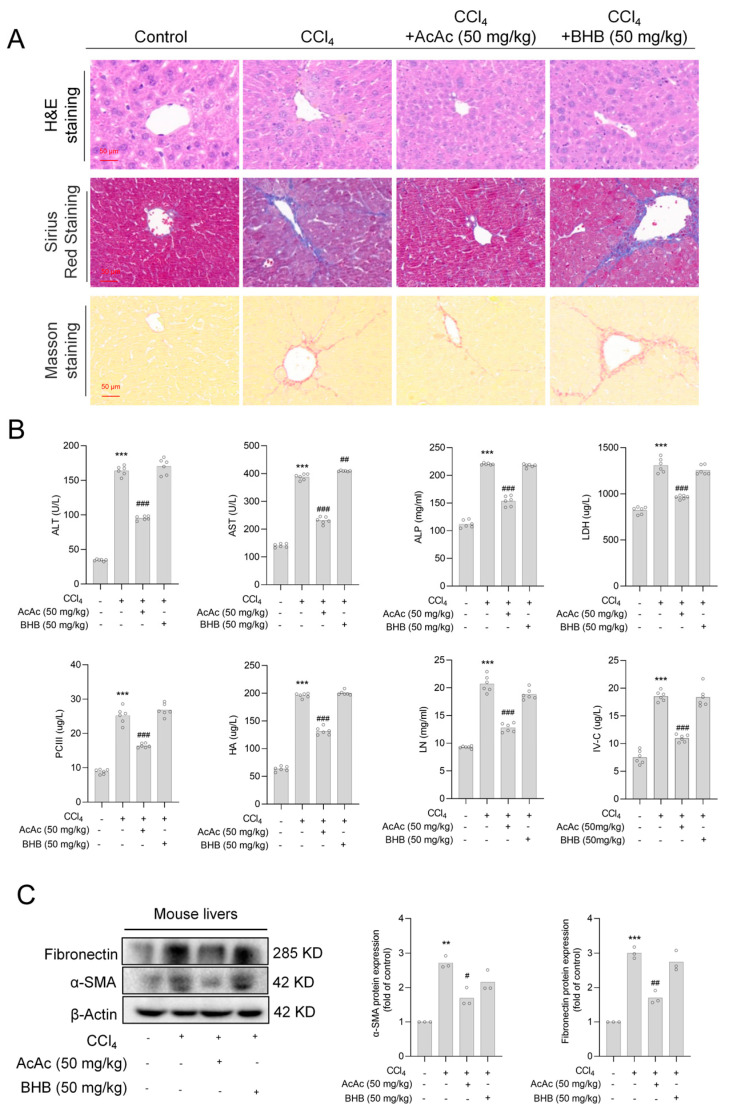
AcAc alleviated HF. (**A**) Following the experiment, the livers of each animal were removed and imaged to determine the form of the liver. Liver sections were stained with hematoxylin and eosin (H&E), Sirius Red, and Masson at 4 μm thickness; scale bar is 50 μm for histopathology research (n = 6 per group). (**B**) Determination of alanine aminotransferase (ALT), aspartate aminotransferase (AST), alkaline phosphatase (ALP), lactate dehydrogenase (LDH), laminin (LN), hyaluronic acid (HA), procollagen III (PC-III), and collagen type IV (IV-C) in mouse serum by ELISA (n = 6 per group). (**C**) Detection of protein expression levels of α-smooth muscle actin (α-SMA) and fibronectin, markers of hepatic stellate cells activation, in mouse liver tissue by Western blot analysis (n = 3 per group). Bars represent the mean ± standard error of the mean (SEM). ** *p* < 0.01, *** *p* < 0.001 vs. vehicle, ^#^ *p* < 0.05, ^##^ *p* < 0.01, ^###^ *p* < 0.001 vs. CCl_4_.

To further verify the consistency of the in vivo and in vitro results, we first induced HF in mice with CCl_4_ for 4 weeks to establish an HF model. The mice were subsequently injected with AcAc (50 mg/kg) or BHB (50 mg/kg) for 3 weeks while continuing CCl_4_ induction. The selected doses of 50 mg/kg for both AcAc and BHB were based on a comprehensive evaluation of several factors, including the pharmacokinetics of the drugs, differences in mouse strains, the objectives of the experiment, and safety considerations, as well as previous studies in the literature [19]. H&E, Masson, and Sirius Red staining showed that CCl_4_-treated mice developed typical HF, which was significantly attenuated by AcAc, whereas BHB did not (Figure 2A). Further serum analysis showed that AcAc had a stronger protective effect against liver injury and HF in vivo (Figure 2B). Subsequently, we extracted proteins from mouse liver tissues and assessed the protein expression of α-SMA and fibronectin (markers of hepatic stellate cells activation) by Western blot analysis. The results showed that AcAc significantly inhibited the expression of HF-related proteins, whereas BHB did not (Figure 2C).

### 2.3. Acetoacetate (AcAc) Increased the Content of Lipid Droplets in Hepatic Stellate Cells (HSCs)

**Figure 3:**
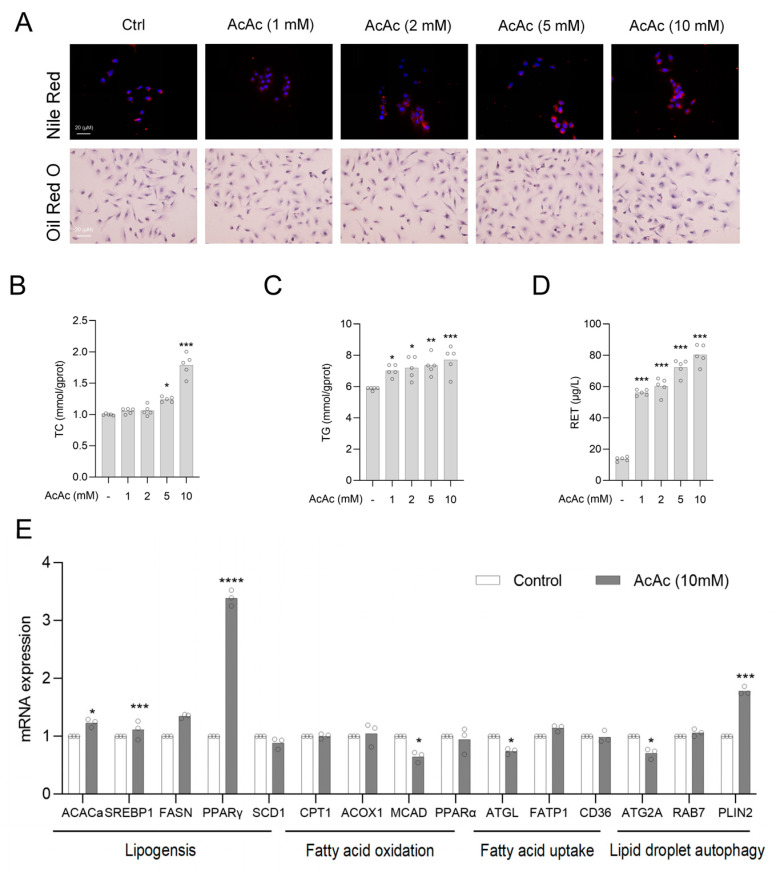
AcAc increased the content of lipid droplets in HSCs. (**A**) Lipid droplet levels of LX-2 cells were detected by Oil Red O staining and Nile Red staining. Scale bars are 20 µm (n = 3 per group). (**B**–**D**) Detection of TG (**B**), TC (**C**), and RET (**D**) levels of LX-2 cells treated with different doses of AcAc by TG, TC, and RET kits (n = 5 per group). (**E**) RT-qPCR was used to measure mRNA levels of lipid metabolism in LX-2 cells treated with AcAc. Lipogenesis: *ACACa*, *SREBP1*, *FASN*, *PPARγ*, *SCD1*; fatty acid oxidation: *CPT1*, *ACOX1*, *MCAD*, *PPARα*; fatty acid uptake: *ATGL*, *FATP1*, *CD36*; lipid droplet autophagy: *ATG2A*, *RAB7*, *PLIN2* (n = 3 per group). Bars indicate the mean ± SEM. * *p* < 0.05, ** *p* < 0.01, *** *p* < 0.001, **** *p* < 0.0001 vs. control.

Lipid droplets (LDs) play a crucial role in maintaining the quiescent state of HSCs. Previous studies have shown that the absence of lipid droplets induces HSCs activation, whereas the restoration of lipid droplets facilitates the shift of activated HSCs back to the resting state [10]. Therefore, we aimed to investigate the effect of AcAc on the lipid droplet content of HSCs. As demonstrated by Oil Red O and Nile Red staining, we found that AcAc significantly increased the accumulation of lipid droplets in LX-2 cells (Figure 3A). Similarly, we further examined the lipid droplet content in AcAc-treated cells using total cholesterol (TC), triglyceride (TG), and Retinol (RET) assay kits, respectively. The results also confirmed that AcAc was able to concentration-dependently increase the levels of the major contents of lipid droplets in LX-2 cells (Figure 3B–D). Lipid droplet metabolism includes the processes of lipogenesis, fatty acid oxidation, fatty acid uptake, and lipid droplet autophagy [20]. We queried the key enzymes involved in lipid droplet metabolism processes through a large body of literature, and the roles of acetyl-CoA acetyltransferase (ACACa), fatty acid synthase (FASN), and sterol regulatory element-binding protein 1 (SREBP1) have extensive validation in lipid synthesis [21], while carnitine palmitoyltransferase 1 (CPT1), acyl-CoA oxidase 1 (ACOX1), and medium-chain acyl-CoA dehydrogenase (MCAD) have been shown to play a role in the oxidation of fatty acids [22]. Fatty acid uptake is accomplished through transporter proteins such as fatty acid transport protein 1 (FATP1) and cluster of differentiation 36 (CD36) [23], while proteins such as perilipin 2 (PLIN2), Ras-related protein rab-7 (RAB7), and autophagy-related protein 2A (ATG2A) regulate the degradation of lipid droplets through mechanisms associated with autophagy [24,25]. We further investigated the effect of AcAc on key proteins involved in lipid droplet metabolism using RT-qPCR. The results indicated that AcAc had minimal impact on the oxidation and uptake phases of lipid droplets. Interestingly, AcAc significantly affected the expression of PPARγ, a critical gene in lipid droplet formation. Additionally, we observed that AcAc increased the expression of PLIN2, an autophagy-related gene. This increase may be attributed to the dual role of PLIN2: it is involved in intracellular lipid droplet formation through chaperone-mediated autophagy (CMA) degradation and plays a key regulatory role in lipid droplet stabilization and storage [26] (Figure 3E). Based on these results, we hypothesized that AcAc may promote the recovery of lipid droplets in activated HSCs by upregulating the expression of PPARγ.

### 2.4. Interference of Peroxisome Proliferator-Activated Receptor Gamma (PPARγ) Reversed the Inhibition of Hepatic Stellate Cells (HSCs) Activation by Acetoacetate (AcAc)

**Figure 4:**
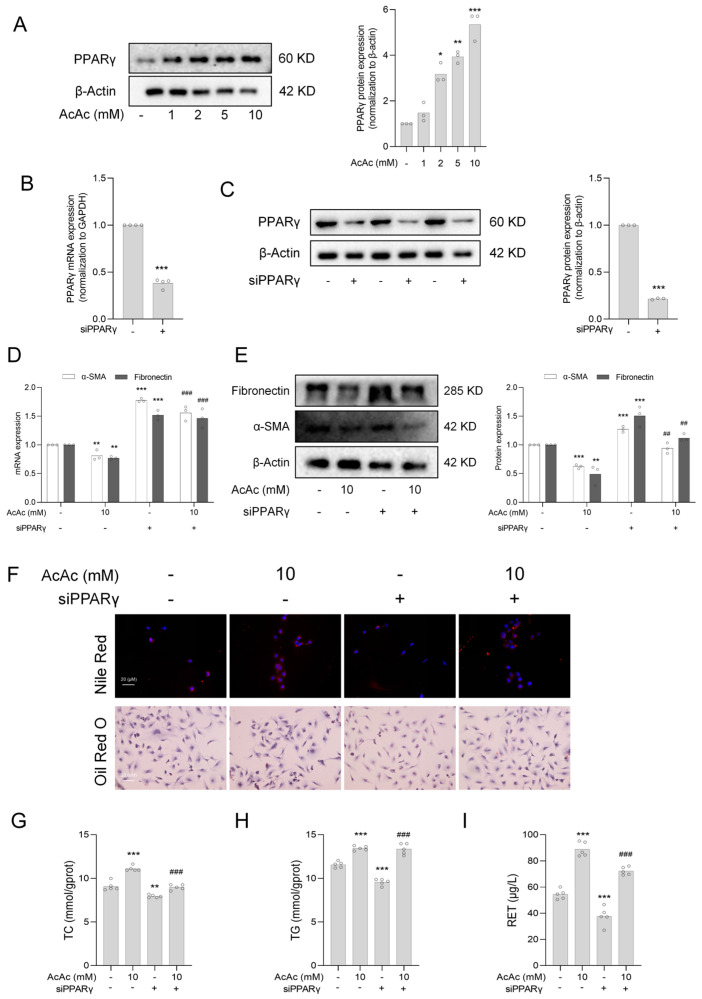
Interference of PPARγ reversed the inhibition of HSC activation by AcAc. (**A**) Protein expression of PPARγ in LX-2 after treatment with different concentrations of AcAc was detected using Western blot analysis (n = 3 per group). (**B**,**C**) The interference efficiency of siPPARγ was verified by RT-qPCR and Western blot analysis (n = 3 per group). (**D**) RT-qPCR was used to detect the mRNA levels of *α-SMA* and *fibronectin* in LX-2 cells transfected with siPPARγ for 24 h after AcAc treatment (n = 3 per group). (**E**) Western blot analysis was used to detect protein levels of α-SMA and fibronectin in LX-2 cells transfected with siPPARγ for 24 h after AcAc treatment (n = 3 per group). (**F**) The content of lipid droplets in LX-2 was detected using Nile Red staining and Oil Red O staining in AcAc-treated LX-2 cells for 24 h and then transfected with siPPARγ for 24 h (n = 3 per group). (**G**–**I**) The levels of TC, TG, and RET in LX-2 cells were determined by treating LX-2 cells with AcAc for 24 h and then transfecting them with siPPARγ for 24 h (n = 3 per group). Bars represent the mean ± standard error of the mean (SEM). * *p* < 0.05, ** *p* < 0.01, *** *p* < 0.001 vs. control, ^##^ *p* < 0.01, ^###^ *p* < 0.001 vs. siPPARγ.

To further investigate the effect of AcAc on PPARγ, we examined the protein expression of PPARγ after the AcAc treatment by Western blot analysis and found that AcAc significantly increased the protein expression of PPARγ (Figure 4A). Next, to demonstrate the important role of PPARγ in the inhibition of hepatic stellate cells activation by AcAc, we used LX-2 cells transfected with siPPARγ to investigate whether it directly affected hepatic stellate cells activation. We detected the interference efficiency of PPARγ by RT-qPCR and Western blot analysis, respectively, and found that small interfering RNA transfection successfully reduced the mRNA and protein expression of PPARγ in LX-2 cells (Figure 4B,C). Therefore, we further experimented and found that the effects of AcAc on α-SMA and fibronectin were suppressed when PPARγ was interfered (Figure 4D,E). In addition, PPARγ interference also reversed AcAc-induced lipid droplet accumulation, specifically, PPARγ interference significantly reversed the increase in lipid droplet content in AcAc-treated LX-2 cells (Figure 4F) and limited the elevation of TC, TG, and RET levels (Figure 4G–H). This suggests that AcAc affects the formation of lipid droplets and the levels of related metabolites by regulating the expression of PPARγ. The above results suggest that PPARγ is an important target of AcAc to inhibit HSCs activation.

### 2.5. Interference of Peroxisome Proliferator-Activated Receptor Gamma (PPARγ) Reversed the Anti-Fibrotic Effect of Acetoacetate (AcAc)

**Figure 5:**
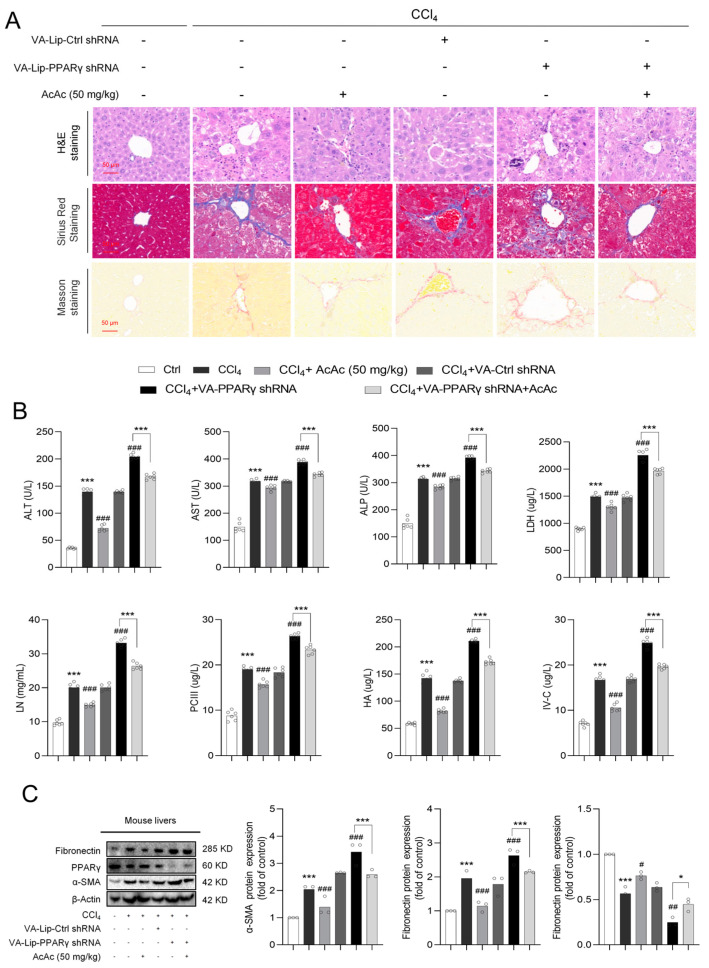
Interference of PPARγ reversed the anti-fibrotic effect of AcAc. (**A**) Following the experiment, the livers of each animal were removed and imaged to determine the form of the liver. Liver sections were stained with hematoxylin and eosin (H&E), Sirius Red, and Masson at 4 μm thickness; scale bar is 50 μm for histopathology research (n = 6 per group). (**B**) Determination of alanine aminotransferase (ALT), aspartate aminotransferase (AST), alkaline phosphatase (ALP), Lactate dehydrogenase (LDH), laminin (LN), hyaluronic acid (HA), procollagen III (PC-III), and collagen type IV (IV-C) in mouse serum by ELISA (n = 6 per group). (**C**) Protein expression levels of α-SMA, fibronectin, and PPARγ in mouse liver tissues detected using Western blot analysis (n = 3 per group). Bars represent the mean ± standard error of the mean (SEM). * *p* < 0.05, *** *p* < 0.001 vs. vehicle, ^#^ *p* < 0.05, ^##^ *p* < 0.01, ^###^ *p* < 0.001 vs. CCl_4_.

To investigate whether AcAc regulates and attenuates HF through the PPARγ, we used mice for intraperitoneal injection of AcAc (50 mg/kg) for 4 weeks and VA-Lip-shRNA-PPARγ was prepared according to a standard reported method and was administered into CCl_4_-challenged mice through the tail vein to reduce the expression of PPARγ [27]. Histological analyses by H&E staining, Masson staining, and Sirius Red staining showed that AcAc treatment significantly improved the liver organization and reduced the manifestation of CCl_4_-induced liver injury and fibrosis. However, when PPARγ was interfered, this protective effect was significantly reversed, and the liver showed renewed damage to its tissue structure and increased fibrosis and inflammation (Figure 5A). Further analysis of the biochemical indices of serum and liver tissue showed that AcAc treatment significantly reduced markers related to HF and liver injury compared with the CCl_4_-treated group; however, the improvement of these indices was significantly attenuated or completely disappeared after PPARγ interference, suggesting that the interference of PPARγ reversed the anti-fibrotic effect of AcAc (Figure 5B). To further verify the critical role of PPARγ in the anti-fibrotic effect of AcAc, the protein expression related to fibrosis was analyzed by Western blot analysis. The results showed that AcAc was able to significantly down-regulate the expression of fibronectin and α-SMA, while up-regulating the level of PPARγ, suggesting that AcAc inhibited hepatic fibrosis by activating PPARγ. However, under the condition of PPARγ interference, the regulatory effect of AcAc on these proteins was significantly weakened, the levels of fibronectin and α-SMA failed to be effectively decreased, and the up-regulatory effect of PPARγ was not maintained (Figure 5C). Taken together, the above experimental results indicate that PPARγ is a key target of the anti-HF effect of AcAc, and the interference of PPARγ can significantly reverse the protective effect of AcAc in alleviating HF. These data reveal the important role of PPARγ in the regulation of HF by AcAc and provide new perspectives for future therapeutic strategies of HF.

## 3. Discussion

Hepatic stellate cells play a crucial role in the onset and progression of HF [1,28]. HSCs have an important function in the maintenance of homeostasis in the liver, typically storing vitamin A and maintaining their resting state via LDs [28]. In response to liver injury, HSCs are activated and transformed into myofibroblasts that secrete large amounts of collagen and other extracellular matrix components, thereby driving HF [6], thus exploring the mechanisms that inhibit the activation of HSCs is critical for the development of effective therapeutic strategies for HF.

PPARγ is a member of the nuclear receptor superfamily, which mainly regulates physiological processes such as lipid metabolism, insulin sensitivity, and inflammatory response [29]. It has been found that PPARγ is not only involved in the regulation of lipid metabolism, but also plays a key role in the immune response and fibrotic process in many diseases [30]. In studies of HF, PPARγ has been shown to have the potential to inhibit the activation of HSCs and attenuate HF [12,13]. PPARγ activates the transcription of downstream genes by binding to its ligands, which in turn regulates biological processes such as lipid droplet production, fatty acid metabolism, and autophagy [31], which may have an impact on the activation of HSCs and the development of HF.

In this study, we revealed the critical role of PPARγ in HF by exploring the effects of AcAc on HSCs. Our data showed that the ketone body AcAc significantly inhibited the activation of HSCs and HF, whereas BHB did not show a similar effect. In vitro experiments, AcAc decreased the expression of α-SMA and fibronectin, markers of HSCs activation, in a concentration-dependent manner and promoted the maintenance of HSCs quiescence by increasing the accumulation of LDs. AcAc inhibited HSCs activation by upregulating the expression of PPARγ, which promotes the generation of LDs and affects the levels of related metabolites. Further in vivo experiments demonstrated that AcAc significantly attenuated the manifestation of liver injury and fibrosis in a mouse model of CCl_4_-induced HF. AcAc suppressed the expression of HF-associated proteins by regulating PPARγ. However, the interference of PPARγ significantly reversed the anti-fibrotic effect of AcAc, suggesting that PPARγ is a key target of the anti-HF effect of AcAc. In summary, AcAc inhibited HSCs activation and HF by activating the PPARγ, and PPARγ interference significantly reversed this effect, suggesting an important role of PPARγ in AcAc regulation of HF. Due to the important role of AcAc in energy metabolism and its short half-life, its degradation does not significantly reduce its effect in inhibiting HSCs activation. This study provides new perspectives for future therapeutic strategies for HF, especially by regulating PPARγ expression.

## 4. Materials and Methods

### 4.1. Chemical Reagents

In vitro reagents: β-hydroxybutyric acid (Cat#HY-113378) was purchased from MedChemExpress (Shanghai, China). Lithium acetoacetate (Cat#3483-11-2) was purchased from Sigma-Aldrich (St. Louis, MI, USA). Primary antibodies against α-SMA (ab32575) and fibronectin (ab2413) were purchased from Abcam Technology (Cambridge, MA, USA). The Proteintech Group (Chicago, IL, USA) supplied the primary antibodies for PPARγ (16643-AP), β-Actin (66009-1-AP), and anti-rabbit IgG (SA00001-2).

In vivo reagents: Acetoacetate (Cat#E808776) was purchased from Mclean (Shanghai, China). Sodium β-hydroxybutyrate (Cat#298360) was obtained from Sigma-Aldrich (St. Louis, MI, USA).

### 4.2. Animal Experiments

The study protocols were sanctioned by the Animal Care and Use Committee of Nanjing University of Chinese Medicine (approval number: 202305A038, Nanjing, China). Male ICR mice, weighing about 18–22 g, were obtained from Hangzhou Medical College. The environment was controlled with a 12-h light/dark cycle, at 22 °C temperature and 40–60% humidity. Following a 7-day acclimation period, HF was induced via intraperitoneal injection of a CCl_4_ and olive oil mixture (1:9 *v*/*v*; 0.5 mL/100 g). ShRNA of mouse PPARγ (5′-GCCCTGGCAAAGCATTTGTAT-3′) was synthesized by Nanjing Jin Ting biological science and technology (Nanjing, China). VA-Lip refers to liposomes that are bound with vitamin A, which are used for targeted delivery of the compound. VA-Lip-shRNA-PPARγ was prepared according to a standard reported method and was administered into CCl_4_-challenged mice through the tail vein [27]. Forty-two mice were randomly assigned to seven groups: Control, CCl_4_, CCl_4_+AcAc (50 mg/kg), CCl_4_+β-hydroxybutyrate (50 mg/kg), CCl_4_+VA-Ctrl shRNA, CCl_4_+VA-PPARγ shRNA, CCl_4_+VA-PPARγ shRNA+AcAc. Except for the control group, the other mice received intraperitoneal injections of the CCl_4_ and olive oil mixture three times a week for eight weeks. After eight weeks, the mice were anesthetized by a 2-min inhalation of isoflurane at a concentration of around 1–1.5%, and blood was collected from their orbits. One portion of the mouse liver was fixed in paraformaldehyde, while the other portion was immediately stored in the −80 °C freezer.

### 4.3. Cell Culture and Transfection

LX-2 cells (a human HSCs line) were obtained from Chemical Book, Shanghai, China. These cells were cultured in Dulbecco’s Modified Eagle Medium (DMEM) supplemented with 10% fetal bovine serum (FBS). Lipofectamine 2000 (Life Technologies Corporation, Carlsbad, CA, USA) was used to transfect siPPARγ plasmids into LX-2 cells according to the manufacturer’s instructions.

### 4.4. Quantitative Real-Time PCR (RT-qPCR)

RNA extraction was performed on LX-2 cells. Next, ABScript II RT Master Mix was used to reverse-transcribe the RNA into cDNA. SYBR Green PCR Fast qPCR Mix (2×, Abclonal, Wuhan, China) was used to amplify cDNA. The mRNA levels of the target genes were determined and normalized to the control gene, GAPDH. Each set of data was independently replicated 3 times. Primer sequences are provided in Table 1.

### 4.5. Western Blotting

Liver or cell lysates were prepared using a RIPA buffer containing protease inhibitors. Tissues or cells were homogenized in a RIPA buffer supplemented with phosphatase inhibitors and protease inhibitors (PMSFs) at a 100:1:1 ratio to minimize protein degradation. Protein concentrations were determined using a NanoDrop spectrophotometer (Thermo Fisher Scientific, Waltham, MA, USA). For Western blot analysis, 50 μg of protein from each sample was loaded onto a 12% polyacrylamide gel for electrophoresis (the gel concentration was adjusted as needed for each experiment). Following electrophoresis, proteins were transferred to a polyvinylidene fluoride (PVDF) membrane, which was subsequently blocked and incubated with the appropriate primary antibody. Protein bands were visualized using enhanced chemiluminescence and quantified. Each experiment was performed with three independent biological replicates. All antibodies used in the study are listed in Table 2.

### 4.6. Oil Red O Staining and Nile Red Staining

Oil Red O staining was performed using the KeyGEN kit (KGA329, KeyGEN, Nanjing, China). After preparing the Oil Red O solution, samples were incubated in it for 15 min. Afterward, LX-2 cells were stained with hematoxylin and slide-mounted. For Nile Red staining (KGDYE22190, KeyGEN), Nile Red was dissolved in DMSO to a concentration of 5 mg/mL. The Nile Red solution was then diluted 1:100 with DMEM medium and applied to a 24-well plate. LX-2 cells were incubated with the Nile Red solution for 10 min at 37 °C, followed by fixation with 4% paraformaldehyde for 30 min. Subsequently, LX-2 cells were incubated with DAPI Dye Solution (KGA215, KeyGEN) for 2 min. All images were captured using a ZEISS Axiovert A1 fluorescence microscope (ZEISS, Baden-Württemberg, Germany). Each set of data was independently replicated 3 times. Representative images showed Oil Red O and Nile Red staining of lipid droplets (100× magnification, scale bar = 20 μm).

### 4.7. Cell Viability Assay

LX-2 cells were harvested and seeded into two 96-well plates at a density of approximately 50–60%. Six replicates were set up for each group. The plates were incubated at 37 °C in a 5% CO_2_ atmosphere for 24 h. The following day, the cells were treated with different concentrations (0–40 mM) of AcAc and BHB, respectively. For the control group, DMSO, which was used to dissolve AcAc and BHB, was added. The cells were then incubated for another 24 h. On the third day, 20 µL of MTT solution (5 mg/mL, 0.5%) was added to each well, and the cells were incubated with the MTT solution for 4 h to allow for the reduction in MTT by metabolically active cells, forming purple formazan crystals. After the incubation, 150 µL of DMSO was added to each well to dissolve the formazan crystals. The absorbance of each well was measured at 490 nm using a Multi-Detection Microplate Reader (Bio-Rad, Hercules, CA, USA). Cell viability was assessed based on the absorbance values.

### 4.8. Biochemical Analysis and Enzyme Linked Immunosorbent Assay

The Retinol (RET) content in LX-2 cells was detected using the RET ELISA kit (YFXEH01860, Yeasen Biotechnology, Shanghai, China). Total cholesterol (TC) and triglyceride (TG) levels in LX-2 cells were measured using the Total Cholesterol Test Kit (A030-2-1) and the Triglyceride Test Kit (A110-2-1). The experiments and data analysis were performed according to the kit instructions. Additionally, blood samples were obtained from mouse orbital veins and allowed to stand at room temperature for 2 h before being centrifuged at 3000 rpm and 4 °C for 30 min. Markers of liver injury and HF: alanine aminotransferase (ALT), aspartate aminotransferase (AST), alkaline phosphatase (ALP), lactate dehydrogenase (LDH), procollagen III (PC-III), hyaluronic acid (HA), laminin (LN), and collagen type IV (IV-C) were analyzed using commercial ELISA kits provided by Nanjing Jinting Biotechnology (Nanjing, China).

### 4.9. Histological Analysis

Tissue samples were sectioned and fixed in 10% neutral buffered formalin. These fixed tissues were then dehydrated and embedded in paraffin, preparing sections 4–6 microns thick. To assess liver morphology and the extent of fibrosis, hematoxylin and eosin (H&E), Masson, and Sirius Red staining were performed. Microscopic images of these stained sections were captured at random using a Zeiss Axio Vert. A1 microscope (ZEISS, Baden-Württemberg, Germany) and a magnification of ×400.

### 4.10. Statistical Analysis

Data are presented as mean ± standard error of the mean (SEM). A Student’s *t*-test was used for comparisons between two groups. One-way and multi-way ANOVA were used to compare more than two groups. Bars represent mean ± SEM. * *p* < 0.05, ** *p* < 0.01, *** *p* < 0.001, **** *p* < 0.0001, ^#^ *p* < 0.05, ^##^ *p* < 0.01, ^###^ *p* < 0.001.

## 5. Conclusions

In summary, our findings provide the first evidence that AcAc inhibits HSCs activation and HF by regulating PPARγ. Future studies need to delve into the role of PPARγ in HF as well as the mechanism of AcAc regulation of PPARγ, which will provide a solid foundation for the prevention and treatment of HF.

## Figures and Tables

**Table 1:** Sequence of primers for RT-qPCR.

Gene	5′ Forward Primer (5′–3′)	3′ Reverse Primer (5′–3′)
Human		
*α-SMA*	CCGACCGAATGCAGAAGGA	ACAGAGTATTTGCGCTCCGGA
*Fibronectin*	AGGAAGCCGAGGTTTTAACTG	AGGACGCTCATAAGTCACC
*GADPH*	ATTCCACCCATGGCAAATTCC	GACTCCACGACGTACTCAGC
*PPARγ*	CATAAAGTCCTTCCCGCTGA	TCTGTGATCTCCTGCACAGC
*ACACa*	TTCTGCACACGTTCCTTGTC	TGCAGCAGCAACACTGAAAT
*SREBP1c*	TGAGGACAGCAAGGCAAAG	CAGGACAGGCAGAGGAAGAC
*FASN*	GTCCACCAGCAACATCAGC	GTTCTCCAGCAAGCCATCTC
*PGC-1*	TCCTTTGGGGTCTTTGAGAA	GGCACGCAATCCTATTCATT
*CPT1a*	GCACATCGTCGTGTACCATC	AATAGGCCTGACGACACCTG
*ACOX1*	CTGTGAGGCACCAGTCTGAA	AGGTGAAAGCCTTCAGTCCA
*MCAD*	ACAGGGGTTCAGACTGCTATT	TCCTCCGTTGGTTATCCACAT
*FATP1*	ACGCGATATACCAGGAGCTG	ATCTTGAAGGTGCCTGTGGT
*CD36*	ACGCTGAGGACAACACAGTCT	GCCACAGCCAGATTGAGAAC
*FAbP1*	GCTGGGTCCAAAGTGATCCA	TGTCACCTTCCAACTGAACCA
*ATGL*	CAACGCCACGCACATCTAC	CAATGAACTTGGCACCAGCC
*SCD1*	TCTAGCTCCTATACCACCACCA	TCGTCTCCAACTTATCTCCTCC
*RAB7*	GTGTTGCTGAAGGTTATCATCCT	GCTCCTATTGTGGCTTTGTACTG
*PLIN2*	TTGCAGTTGCCAATACCTATGC	CCAGTCACAGTAGTCGTCACA
si*PPARγ*	GGAUGCAAGGGUUUCUUCCTT	GGAAGAAACCCUUGCAUCCTT
Mouse		
GADPH	GGAGAGTGTTTCCTCGTCCC	ACTGTGCCGTTGAATTTGCC
*α-SMA*	GTACCACCATGTACCCAGGC	GCTGGAAGGTAGACAGCGAA
*Fibronectin*	ATGTGGACCCCTCCTGATAGT	GCCCAGTGATTTCAGCAAAGG
*PPARγ*	GGAAGACCACTCGCATTCCTT	GTAATCAGCAACCATTGGGTCA

**Table 2:** Antibodies used in this study.

Antibodies	Host	Dilution	Catalogue	Company
anti-β-Actin	Rabbit pAb	1:2000–1:3000	66009-1-AP	Proteintech
anti-*α*-SMA	Rabbit pAb	1:1000–1:4000	ab32575	Abcam
anti-Fibronectin	Rabbit pAb	1:1000–1:4000	ab2413	Abcam
anti-PPARγ	Rabbit pAb	1:500–1:3000	A6981	ABclonal
anti-rabbit IgG	Rabbit pAb	1:500–1:2000	SA00001-2	ABclonal

## Data Availability

The data that support the findings of this study are available from the corresponding author upon reasonable request.

## Abbreviations

The following abbreviations are used in this manuscript:

HF	Hepatic fibrosis
HSCs	Hepatic stellate cells
ECM	Extracellular matrix
α-SMA	α-smooth muscle actin
KD	Ketone body
AcAc	Acetoacetate
BHB	beta-hydroxybutyric acid
LDs	lipid droplets
TG	Triglyceride
TC	Total Cholesterol
RET	Retinol
PPARγ	Peroxisome proliferator-activated receptor γ
OXCT1	3-oxoacid CoA-transferase 1
ACACa	Acetyl-CoA Acetyltransferase 1
FASN	Fatty Acid Synthase
SREBP1	Sterol Regulatory Element-Binding Protein 1
CPT1	Carnitine Palmitoyltransferase 1
ACOX1	Acyl-CoA Oxidase 1
MCAD	Medium-Chain Acyl-CoA Dehydrogenase
FATP1	Fatty Acid Transport Protein 1
CD36	Cluster of Differentiation 36
PLIN2	Perilipin 2
RAB7	RAB7—Ras-Related Protein Rab-7
ATG2A	Autophagy-Related Protein 2A
CMA	Chaperone mediated autophagy
ALT	Alanine aminotransferase
AST	Aspartate aminotransferase
ALP	Alkaline phosphatase
LDH	Lactate dehydrogenase
LN	Laminin
HA	Hyaluronic acid
PC-III	Procollagen III
IV-C	Collagen type IV
